# Pediatric Posterior Reversible Encephalopathy Syndrome Secondary to Post-Streptococcal Glomerulonephritis: A Case Report

**DOI:** 10.7759/cureus.38996

**Published:** 2023-05-14

**Authors:** Ganaraja V Harikrishna, Jayashankar CA, Amey Joshi, Aman Shuhab, Suresha Kodapala

**Affiliations:** 1 Neurology, Vydehi Institute of Medical Sciences & Research Center, Bangalore, IND; 2 Internal Medicine, Vydehi Institute of Medical Sciences & Research Centre, Bangalore, IND; 3 Internal Medicine, Vydehi Institute of Medical Sciences and Research Centre, Bangalore, IND; 4 Neurology, Vydehi Institute of Medical Sciences and Research Centre, Bangalore, IND

**Keywords:** hypertension in young patients, neuro radiology, pediatric case, posterior reversible encephalopathy syndrome (pres), post-streptococcal glomerulonephritis

## Abstract

This case report presents a unique case of a 15-year-old male with post-streptococcal glomerulonephritis (PSGN) who developed posterior reversible encephalopathy syndrome (PRES). The patient presented with symptoms of fever, headache, emesis, visual disturbances, and involuntary movements of all four limbs. On examination, the patient had elevated blood pressure, decreased visual acuity of the left eye, leukocytosis, and uremia. MRI findings showed symmetrical enhancement of superficial and deep watershed areas, predominantly in the occipital and temporal regions. Treatment with antibiotics and anti-hypertensives resulted in the complete resolution of hyperintense lesions seen in brain MRI after three weeks, and the patient remained symptom-free for one month. This case highlights the rare association between PSGN and PRES and emphasizes the importance of monitoring and managing hypertension in patients with PSGN. Understanding the association between these two conditions may lead to earlier diagnosis and treatment of PRES, ultimately improving patient outcomes.

## Introduction

Posterior reversible encephalopathy syndrome (PRES) is a rare neurological disorder that can result in a variety of clinical manifestations, including seizures, altered consciousness, headaches, and visual disturbances. Although the underlying pathophysiology of PRES is not fully understood, hypertension, immunosuppressive therapy, and renal dysfunction have been suggested as contributing factors [1]. Here, we present the unique case of a 15-year-old male with post-streptococcal glomerulonephritis (PSGN) who developed PRES.

While PRES has been reported in association with various conditions, there have been only a few cases documenting the association between PSGN and PRES [2,3]. This case underscores the importance of monitoring and managing hypertension in patients with PSGN, particularly those at higher risk for complications. The association between PSGN and PRES is intriguing and warrants further investigation, as it may provide important insights into the underlying mechanisms of PRES. Understanding the association between these two conditions may lead to earlier diagnosis and treatment of PRES, ultimately improving patient outcomes.

## Case presentation

A 15-year-old male presented to our department with ongoing symptoms of fever and headache for seven days. The boy also presented with frequent episodes of projectile emesis during this period. He also reported blurry vision in both eyes that lasted for 24 hours and resolved gradually. The history was significant for two observed episodes of involuntary movements of all four limbs that lasted three to five minutes and were associated with postictal confusion. Past medical history was unremarkable for previous similar episodes, surgeries, or substance abuse, and the boy was up to date with his vaccinations as per the National Immunization Schedule of India.

On examination, the boy was found to have a blood pressure of 150/100 mmHg (99th percentile as per the Indian Pediatric Association) and a heart rate of 82 beats/minute. Neurological examination was unremarkable except for decreased visual acuity of the left eye. Fundoscopy was unremarkable. Investigations were significant for leukocytosis, positive IgM antibodies for Dengue, decreased complement 3, elevated anti-streptolysin (ASO) titers, uremia, hematuria, and proteinuria (Table 1).

**Table 1:** Laboratory investigations Hb: hemoglobin; AST: aspartate transferase; ALT: alanine transferase; ALP: alkaline phosphatase; aPTT: activated partial thromboplastin time; CSF: cerebrospinal fluid; ANA: antinuclear antibody; HBsAg: hepatitis B surface antigen; HIV: human immunodeficiency virus; ELISA: enzyme-linked immunosorbent assay; PS: peripheral smear, * 3 smears

Laboratory Investigations	Day 1	Normal Range
Hb (g/dl)	13.7	12.4-16.4
Total count (µL)	17.7	4.8-10.8
Packed cell volume (%)	41.8	30-44
Mean corpuscular volume (fL)	82.5	80-96
Mean corpuscular hemoglobin (pg)	27.1	27-31
Platelet count (µL)	390000.0	150000-450000
Blood urea (mg/dl)	47.9	7-20
Serum creatinine (mg/dl)	0.7	0.45-0.81
Total bilirubin (mg/dl)	0.6	0.1-1.2
Direct bilirubin (mg/dl)	0.1	<0.3
Total protein (g/dl)	6.4	6-8.3
Albumin (mg/dl)	3.6	3.4-5.4
AST (IU/L)	13.0	10-40
ALT (IU/L)	15.0	10-40
ALP (IU/L)	14.6	<350
Globulin (g/dl)	2.8	2-3.5
Lactate dehydrogenase (units/L)	338.0	140-280
Prothrombin time (seconds)	12.2	11-13.5
International normalized ratio	1.1	0.8-1.1
aPTT (seconds)	26.6	21-35
Serumsodium (mEq/L)	134.0	135-145
Serum potassium (mEq/L)	4.9	3.4-4.7
Serum chloride (mEq/L)	100.7	98-106
Serum calcium ^C^ (mg/dl)	9.2	9-10.5
C-reactive peptide (mg/dl)	0.1	<0.9
CSF Cell count (per cumm)	1.0	<5
CSF Cell type	100% lymphocytes	-
CSF sugar	54.7	50-80
CSF protein	36.7	15-45
CSF meningoencephalitis panel	Negative	-
Serum complement 3 (C3)	9.6	79-152
Serum complement 4 (C4)	29.3	16-38
Anti-streptolysin titres	192.0	-
ANA	negative	-
Urine micro total protein (mg/dl)	222.5	0-14
Urine protein creatinine ratio (mg/mg)	3.1	<0.2
Urine leukocyte	25.0	0-5
Urine RBCs	Plenty	<4
HBsAg	Non-reactive	-
HIV 1/2 by ELISA	Non-reactive	-
Rapid Plasma Reagin	Non-reactive	-
Dengue NS1	Negative	-
Dengue IgG	Negative	-
Dengue IgM	Positive	-
PS for malarial parasite*	negative	-

**Figure 1:**
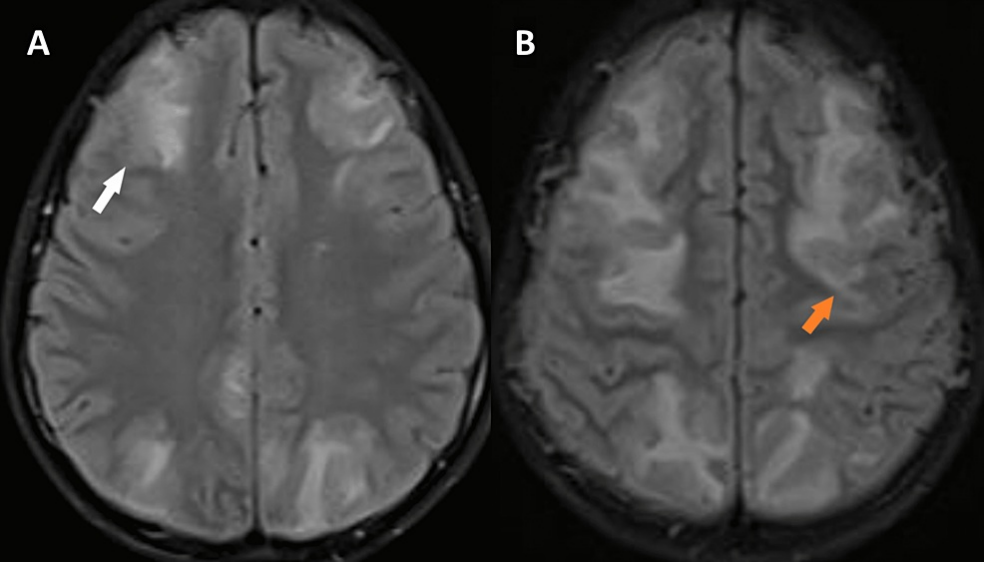
Brain magnetic resonance imaging FLAIR sequence at presentation Brain MRI FLAIR sequence showing symmetrical hyperintensities in both fronto-parietal-occipital cortex and subcortical region involving superficial (A-white arrow) and deep watershed regions (B-orange arrow). FLAIR: fluid-attenuated inversion recovery

Electrocardiogram, echocardiography, chest X-rays, and cerebrospinal fluid (CSF) studies were unremarkable. MRI findings were remarkable for the symmetrical enhancement of superficial and deep watershed areas, predominantly in the occipital and temporal regions (Figure 1).

The results of 72 hours of blood pressure (BP) monitoring revealed elevated blood pressure with systolic readings > 140mmHg with the highest recorded blood pressure of 150/100 mmHg (99th percentile as per the Indian Pediatric Association). Considering the recent history of fever and the findings of elevated ASO titers, decreased C3, proteinuria, and hematuria, we diagnosed our patient with post-streptococcal glomerulonephritis. Furthermore, the MRI findings of the brain and the history of elevated blood pressure were confluent with the diagnosis of PRES.

**Figure 2:**
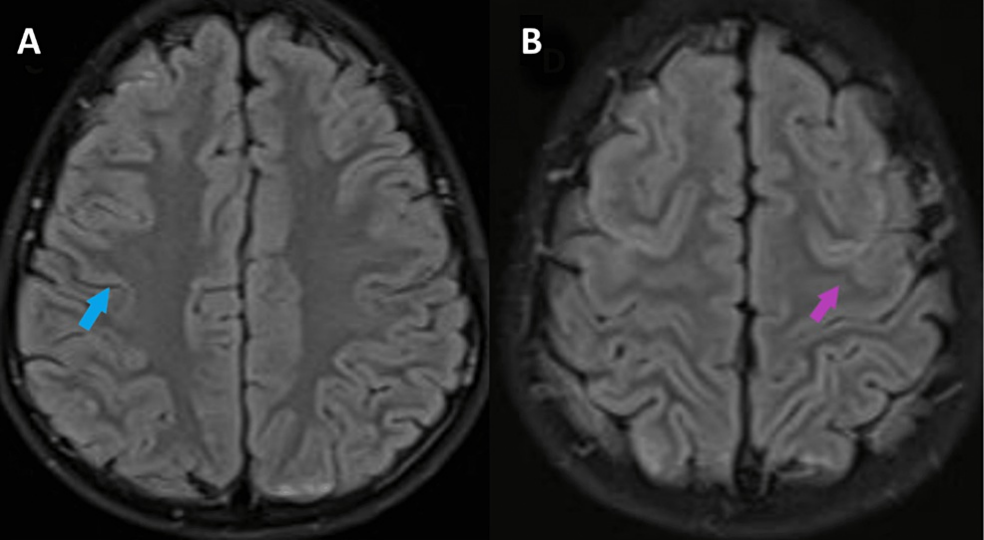
Repeat brain magnetic resonance imaging-FLAIR sequence three weeks after presentation Repeat brain MRI-FLAIR sequence showing resolution of hyperintensities noted on initial imaging (A-blue arrow, B-violet arrow) FLAIR: fluid-attenuated inversion recovery

Our patient was treated conservatively with ceftriaxone for antimicrobial coverage and amlodipine for blood pressure management. Six days after admission and treatment, the boy was asymptomatic, however, with minimal hematuria. A repeat MRI after three weeks revealed a complete resolution of hyperintense lesions (Figure 2).

His seizure-like symptoms, vomiting, and blurry vision had not recurred. He remains symptom-free for one month.

## Discussion

PRES is a rare but important clinical entity that has been increasingly recognized over the past few decades. It is a clinical and radiological syndrome characterized by headache, altered mental status, seizures, and visual disturbances and is typically associated with hypertension [4]. Although PRES is generally considered to be more common in adults, it can also occur in children, albeit much less frequently. The present case of PRES secondary to PSGN is particularly unique and important because it highlights a rare association between these two conditions.

The prevalence of PRES in childhood is estimated to be less than 5% of all cases of PRES. While hypertension is a common predisposing factor for PRES in adults, it is less common in children. In children, the most common underlying etiologies for PRES are infections, autoimmune disorders, and renal disease. Additionally, the clinical presentation of PRES in children may differ from that in adults, with children more likely to present with seizures and altered mental status [2]. The approach to the management of PRES in children differs from adults in that careful attention must be paid to the potential long-term neurocognitive and developmental outcomes [2].

The pathophysiology of PRES is not well understood, but it is thought to involve the disruption of the blood-brain barrier, leading to vasogenic edema in the posterior regions of the brain. The underlying mechanisms that cause PRES are thought to be related to hypertension, renal disease, autoimmune disorders, and exposure to certain medications [5]. In the setting of hypertension, elevated blood pressure may lead to endothelial dysfunction and disruption of the blood-brain barrier. Renal disease may lead to electrolyte imbalances and hypertensive encephalopathy, while autoimmune disorders may lead to immune-mediated endothelial damage. Exposure to certain medications, such as chemotherapeutic agents, immunosuppressants, and calcineurin inhibitors, has also been associated with PRES. The rarity of PRES in children may be due to the differences in the anatomy and physiology of the pediatric brain, which may make it more resistant to injury [2].

The management of PRES involves the identification and treatment of the underlying cause, along with supportive care. In cases of PRES related to hypertension, blood pressure control is crucial to prevent further cerebral edema. In cases related to renal disease, renal function should be closely monitored and electrolyte imbalances corrected. Supportive care includes seizure prophylaxis and the management of any neurological symptoms or complications. In severe cases, such as those with refractory seizures or increased intracranial pressure, more aggressive interventions may be necessary, including intubation, mechanical ventilation, and neurosurgical intervention [4].

The outcomes of PRES vary depending on the severity of the disease and the underlying cause. In most cases, the symptoms of PRES resolve within days to weeks, and the radiological abnormalities resolve within months. However, long-term neurocognitive and developmental outcomes in children with PRES are not well understood. A recent study found that children with PRES had a good overall prognosis, with most patients showing complete or near-complete recovery. However, the study also found that patients with underlying renal disease had a higher risk of poor outcomes [6].

## Conclusions

In conclusion, the present case of PRES in a patient with PSGN is a rare and important finding that adds to our understanding of the diverse etiologies and clinical presentations of PRES. This case highlights the importance of prompt recognition and treatment of PRES, particularly in children, and underscores the need for further research to elucidate the underlying mechanisms and optimal management strategies for this condition.
